# A Multi-Node Detection Algorithm Based on Serial and Threshold in Intelligent Sensor Networks

**DOI:** 10.3390/s20071960

**Published:** 2020-03-31

**Authors:** Guanghua Zhang, Zonglin Gu, Qiannan Zhao, Jingqiu Ren, Shuai Han, Weidang Lu

**Affiliations:** 1 School of Electrical Engineering and Information, Northeast Petroleum University, Daqing 163318, China; dqzgh@nepu.edu.cn (G.Z.); guzonglingzl@163.com (Z.G.); zhaoqiannan824@163.com (Q.Z.); 2 Communication Research Center, Harbin Institute of Technology, Harbin 150080, China; hanshuai@hit.edu.cn; 3 College of Information Engineering, Zhejiang University of Technology, Hangzhou 310014, China; luweid@zjut.edu.cn

**Keywords:** intelligent sensor, message passing algorithm, threshold, serial, complexity

## Abstract

With the continuous progress of science and technology, intelligent wireless sensor network (IWSN) communication has become indispensable in its role in production and life because of its convenient network settings and flexible use. However, with the widespread availability of intelligent wireless sensor networks, the use of many wireless sensor nodes constitutes a multi-node wireless communication system, which turns the accuracy and low complexity of multi-node detection in sensor networks into a problem. Although the traditional algorithm has excellent performance, it cannot give consideration to both accuracy and complexity. Therefore, a maximum logarithm message passing algorithm based on serial and threshold (S-T-Max-log-MPA) for multi-mode detection in IWSN is proposed in this paper. In this algorithm, the threshold is used to determine the necessary conditions of sensor node stability first, and then the sensor node information updating is integrated into the resource node information updating, so that the system can maintain good accuracy, performance, and change the situation of poor system accuracy at low threshold. Compared with the traditional algorithm, the proposed algorithm significantly changes the algorithm complexity reduction rate of the system multi-node detection. Simulation results show that the algorithm has a good balance between accuracy and complexity reduction rate.

## 1. Introduction

The intelligent wireless sensor network (IWSN) is composed of a large number of static or mobile micro sensor nodes; the nodes form a wireless communication system [1]. Intelligent sensor networks are widely used in military, traffic, environmental monitoring, medical and other aspects because of their flexible network settings and simple equipment location modification [2,3]. The rapid development of communication networks and intelligent sensor technology has resulted in large-scale growth of sensor networks [4]. The use of many sensor nodes makes the demand for large-scale link, low delay and high capacity of the communication network to sharply increase [5,6,7]. Therefore, how to detect the existence of user nodes from many network nodes has become one of the research topics.

With the rapid development of fifth generation (5G) mobile communication systems, 5G has become an important research objective in the development of communication networks and intelligent sensor networks [8,9]. 5G has a throughput improvement of about 25 times higher than 4G, and a resource utilization efficiency of more than 10 times [10]. In addition, 5G also includes key technologies such as large-scale antenna arrays, ultra-dense networks, millimeter wave communication, non-orthogonal multiple access and new network architecture, etc. [11,12,13,14]. Therefore, it can well meet the needs of current communication networks and wireless sensor networks. Sparse code multiple access (SCMA) is a new multiple access technology of 5G [15]; it synthesizes the ideas of code division multiple access (CDMA) and orthogonal frequency division multiple access (OFDMA) [16], realizes the frequency domain non-orthogonal multiple access (NOMA) [17], and provides a reliable technical method for multiuser access and detection in 5G application. SCMA has the characteristics of large capacity, low delay, multi-connection and strong anti-multipath ability [18,19,20], which can better meet the needs of 5G for higher spectral efficiency [21,22]. SCMA technology is an improvement over the basis of low density multiple access technology; its application greatly improves the performance of multi-user access and detection [23,24]. In order to get the codebook, SCMA technology combines spread spectrum technology and constellation mapping technology to realize the operations of displacement, rotation and conjugation of high-dimensional constellation [25,26,27,28,29]. When the SCMA technology is used in IWSN, the following steps are adopted to realize the processing of the sensor nodes at the sending end: (i) The sparse spread spectrum sequences are assigned to every sensor node. (ii) The sparse spread spectrum technology is applied to sparse the data of sensor nodes. (iii) The data of all sensor nodes are mapped into an n-dimensional codebook through a multi-dimensional constellation, and then these data are superimposed on the same time-frequency resources of each transmission layer. (iv) These data are sent to the same channel for transmission. The sensor nodes can get better mapping gains with this scheme [30,31]. At the receiving end, the message passing algorithm (MPA) decoder is used for multi-user detection, and then the received signal, mapping mode and channel coefficient are used to decode the signal. However, when the number of sensor nodes in SCMA system is much larger than the number of resource nodes, the complexity of the MPA algorithm will be very high.

The application of 5G not only brings high-speed data transmission, but also promotes the development of multiple access and detection technology, such as the original MPA, the MPA based threshold (T-MPA) and the MPA based serial (S-MPA). However, the existing multi-node detection algorithm still cannot solve the problem of low transmission accuracy and high algorithm complexity in wireless sensor networks, so how to reduce the algorithm complexity and improve the message transmission accuracy is the key research objective in IWSN. In reference [32], a direct sequence code division multiple access (DS-CDMA) based communication system is proposed, which becomes the wireless interface of an integrated sensor micro system and improves the limitation of power and area in the communication system of a miniaturized sensor network. In reference [33], a heuristic CDMA multiuser detection scheme based on the harmony search (HS) algorithm is proposed. The multi-user detection scheme based on the harmony search (HS) algorithm reduces the complexity of the algorithm by searching a group of nearly optimal candidate vectors, but it still has high algorithm complexity and bit error ratio (BER). In reference [34], a threshold based MPA algorithm is proposed, which uses a threshold to control the algorithm, so as to reduce the complexity of the detection algorithm. An MPA algorithm based on a serial strategy is proposed in [35]. In reference [36], an improved serial scheduling based MPA (ISS-MPA) detection scheme is proposed. The maximum number of new message updating in the corresponding factor graph is used to select the scheduling order of user nodes, so as to maintain good BER performance and reduce the complexity of the detection algorithm. Although the above algorithms have obvious changes in the algorithm complexity and transmission accuracy compared with the traditional multi-node passing algorithm, the disadvantages of high algorithm complexity and low transmission accuracy still exist simultaneously in IWSN.

In the multi-node detection algorithm, the original MPA has good performance and low complexity, but because of the exponential (EXP) algorithm, the complexity of the multi-node detection algorithm is very high. The approximate and the maximum calculation are used in the maximum logarithm message passing algorithm (Max-log-MPA), which results in the loss of some information and poor system accuracy. The T-MPA algorithm adopts the hard decision mechanism, which reduces the complexity of the system algorithm, but has the problem of high accuracy when the threshold is low. The sensor node information updating is integrated into the resource node information updating in the S-MPA algorithm, which effectively reduces the complexity of the system multi-node detection algorithm, but the transmission accuracy of sensor node information is poor. Therefore, in order to further improve the transmission accuracy and complexity of the multi-node detection algorithm in the IWSN, a maximum logarithm message passing algorithm based on serial and threshold (S-T-Max-log-MPA) for multi-mode detection in IWSN is proposed in this paper. In this algorithm, the exponential operation is reduced to the additive operation by using the operation of the logarithmic field, and by reducing the storage space of the sensor node codebook. The serial algorithm and threshold algorithm are used to reduce the code storage time in the iterative process, improve the accuracy of information transmission of sensor nodes, reduce the message loss in the updating process, and reduce the number of iterations of sensor nodes. Therefore, the proposed algorithm in this paper reduces the computational complexity to a large extent while maintaining good accuracy of the wireless sensor network system, especially improving the poor accuracy of data transmission at low threshold in IWSN.

## 2. SCMA System Model

**Figure 1:**
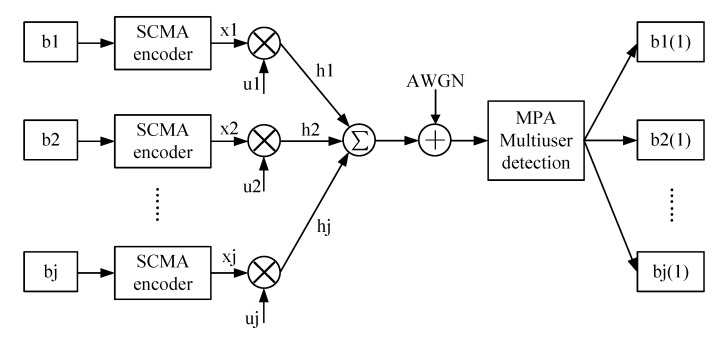
Uplink sparse code multiple access (SCMA) communication system model.

The SCMA uplink model consists of transmitter, transmission channel and receiver, as shown in Figure 1. Assuming that the number of sensor nodes is j and the number of time-frequency resource blocks is K (J>K), the binary bit data stream b_j_ (b_1_, b_2_, …, b_J_) is obtained after the sensor node information u_j_ (u_1_, u_2_, …, u_J_) is encoded by the source and the channel. Then data enters the SCMA encoder, which maps it to the sparse SCMA codebook x=f(b_j_); the mapping process of SCMA [37,38] can be defined as $f:B^{log_{2}M}\to \chi$. B is the set of binary numbers, χ is the sensor node’s codebook, and M is the size of the codebook. Due to the channels of each layer of the upper link being different, the channel factor is different, here assuming that the channel factor is h_j_ (h_1_, h_2_, …, h_J_).

The size M of the codebook depends on the number of bits of binary data. Sensor node data is divided into several groups according to a-bit. For a-bit user data, the size M of the codebook is shown in Equation (1):

(1) $$M=2^{a}.$$

The ratio of the number of sensor nodes that can be carried on a certain time-frequency resource block and the size of a codebook in the SCMA system is defined as the overload factor, and its calculation method can be expressed as Equation (2):

(2) $$\lambda =\frac{J}{M}.$$

**Figure 2:**
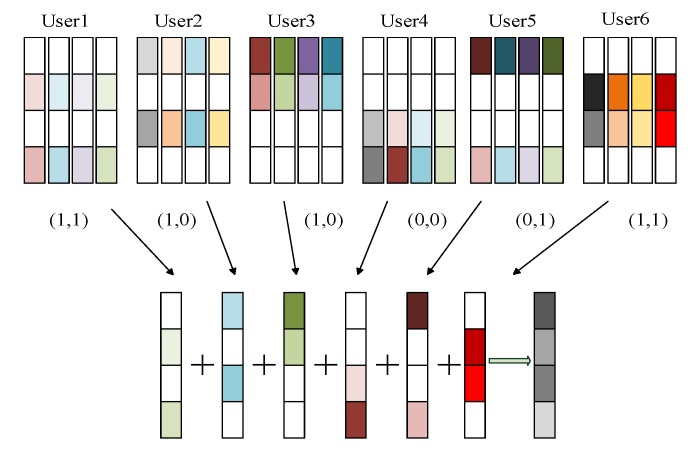
SCMA coding principle.

If six sensor nodes occupy four time-frequency resource blocks, the overload ratio is 150%. As shown in Figure 2, the bit information of six sensor nodes is mapped to the codewords of different codebooks [39], and each sensor node has a unique codebook. Each codebook contains two non-zero elements and two zero elements, so the codebook length is 4. In the factor graph, 1 represents the non-zero element and 0 represents the zero element, then the user information can be expressed by a matrix as in Equation (3):

(3) $$F_{4\times 6}=\left[\begin{array}{cccc} \begin{array}{c} 0\\ 1\\ 0\\ 1 \end{array} & \begin{array}{cc} \begin{array}{cc} \begin{array}{c} 1\\ 0\\ 1\\ 0 \end{array} & \begin{array}{c} 1\\ 1\\ 0\\ 0 \end{array} \end{array} & \begin{array}{c} 0\\ 0\\ 1\\ 1 \end{array} \end{array} & \begin{array}{c} 1\\ 0\\ 0\\ 1 \end{array} & \begin{array}{c} 0\\ 1\\ 1\\ 0 \end{array} \end{array}\right].$$

The K resource blocks are loaded on the subcarrier for transmission, and the signals on the K subcarrier at the receiving end are expressed as Equation (4):

(4) $$y=\sum_{j=1}^{J}\mathrm{diag}(h_{j})x_{j}+n,$$

where, x_j_ = [x_1,j_, x_2,j_, …, x_K,j_]^T^ is the k-dimension SCMA code of sensor node j, and the non-zero element is K. The SCMA code x_j_ has sparsity, which can reduce the codeword conflict at time-frequency resource k. The vector h_j_ = [h_1_, h_2_, …, h_K_]^T^ is the receiver channel factor vector. The vector n_j_ = [n_1_, n_2_, …, n_K_]^T^ is the White Gaussian Noise vector added to the channel with distribution *N*(*0*, σ^2^*I*) [40]. The vector y_j_ = [y_1_, y_2_, …, y_K_]^T^ is the signal received by the receiver.

## 3. MPA Algorithm

The MPA algorithm is the main detection algorithm of the SCMA system [41]; it updates information of sensor nodes and resource nodes by a factor graph [42]. In this paper, the sensor node is regarded as the variable method node VN, and the resource block is regarded as the function node FN. In the t-th iteration, function node c_k_ sends the information of variable node u_j_, expressed as $I_{k\to j}^{t}$ On the contrary, the information of function node u_j_ sent by variable node c_k_ in the t-th iteration is expressed as $I_{j\to k}^{t}$. If t_max_ is the maximum number of iterations, and t is the number of iterations, when t = t_max_, the symbol probabilities of information transmitted by sensor nodes are calculated respectively [43].

Step 1: Update the information of function node FN, as shown in Equations (5) and (6):

(5) $$I_{c_{k}\to u_{j}}^{0}(x_{j})=\frac{1}{M},$$

(6) $$I_{c_{k}\to u_{j}}^{t}(x_{j})=\sum_{∼x_{j}}\left\{\frac{1}{\sqrt{2\pi \delta }}\exp{\left(-\frac{1}{2\delta^{2}}\left\|y_{k}-\sum_{v\in \xi_{k}}h_{k,v}x_{k,v}\right\|\times \prod_{m\in \xi_{k}/j}I_{c_{m}\to u_{k}}^{t-1}(x_{j})\right)}^{2}\right\},$$

where, $∼\left\{x_{j}\right\}$ represents the edge probability of symbol $x_{j}$, $\xi_{k}/j$ represents the set of all variable nodes in VN that are connected to the function nodes c_k_ except the j-th sensor node.

Step 2: Update the VN information of the variable node, as shown in Equation (7):

(7) $$I_{u_{j}\to c_{k}}^{t}(x_{j})=\prod_{m\in \xi_{j}/k}I_{u_{k}\to c_{m}}^{t}(x_{j}),$$

where, $\xi_{j}/k$ represents the collection of all function nodes in FN that are connected to the variable node x_j_ except the k-th function node.

Step 3: When the maximum number of iterations is reached, the symbol output probability after MPA decoding is shown in Equation (8):

(8) $$Q(x_{j})=\prod_{k\in \xi_{j}}I_{c_{k}\to u_{j}}^{t_{max}}(x_{j}).$$

## 4. S-MPA Algorithm

The S-MPA algorithm is improved on the basis of the original MPA, which uses serial updating of the resource nodes. In the process of message iteration, the sensor node message updating is integrated into the resource node information updating, and the updated information is delivered immediately, which reduces the storage process of intermediate variables and improves the convergence speed of the messages compared with the original MPA algorithm.

The resource node message delivery of the S-MPA algorithm is shown in Equations (9)–(11):

(9) $$I_{c_{k}\to u_{j}}^{t}(x_{j})=\sum_{∼x_{j}}\left\{\frac{1}{\sqrt{2\pi \delta }}\exp{\left(-\frac{1}{2\delta^{2}}\left\|y_{k}-\sum_{v\in \xi_{k}}h_{k,v}x_{k,v}\right\|\right)}^{2}\times \frac{{\left[Q^{t-1}(x_{j})\right]}^{new}}{I_{c_{m}\to u_{k}}^{t}(x_{j})}\times \prod_{m\in \xi_{k}/j}\frac{{\left[Q^{t-1}(x_{j})\right]}^{old}}{I_{c_{m}\to u_{k}}^{t-1}(x_{j})}\right\},$$

(10) $${\left[Q^{t-1}(x_{j})\right]}^{old}=\prod_{m\in \xi_{k}/j}I_{c_{m}\to u_{k}}^{t-1}(x_{j}),$$

(11) $${\left[Q^{t-1}(x_{j})\right]}^{new}=\frac{{\left[Q^{t-1}(x_{j})\right]}^{old}}{I_{c_{m}\to u_{k}}^{t-1}(x_{j})}*I_{c_{m}\to u_{k}}^{t}(x_{j}),$$

where t is the number of iterations: i≠j, $i\in \xi_{k}$, $j\in \xi_{j}$, $\xi_{k}$ and $\xi_{j}$ represent the set of 1 positions in row K and column j of the factor graph matrix F respectively; $x_{v,k}$ represents the codeword of the v-th sensor node on the k-th resource block; and $h_{k,v}$ represents the channel coefficient of the v-th sensor node on the k-th resource block. The terms [ ]^new^ and [ ]^old^ represent the code word probabilities of the sensor node after and before the update.

From Equations (9) and (10), we can see that the confidence of ${\left[Q^{t-1}(x_{j})\right]}^{old}$ is lower than that of ${\left[Q^{t-1}(x_{j})\right]}^{new}$.

## 5. S-T-Max-log-MPA Algorithm

In order to reduce the complexity of the detection algorithm, the S-T-Max-log-MPA is proposed. Based on the Max-log-MPA algorithm [44], this proposed algorithm introduces the serial updating algorithm and threshold application. In the Max-log-MPA algorithm, the exponential algorithm is changed into the process of sum algorithm and maximum value. The sensor node information updating is integrated with the resource node information updating in the S-MPA algorithm, which reduces the complexity of information storage. In the T-MPA algorithm, a hard decision is used to effectively reduce the sensor node information that needs to be updated in each cycle [45]. The algorithm proposed here is based on the advantages of the above algorithms; it can effectively reduce the complexity of the detection algorithm while maintaining a good BER.

The basic idea of this algorithm is as follows: the threshold is added based on the S-MPA algorithm, and the codeword reliability and sensor node stability are combined as the index to judge the codeword reliability of sensor nodes. Before the message updating, the stability of the sensor variable node is judged. If the log likelihood ratio (LLR) of the sensor variable node meets the threshold condition, the sensor variable node is decoded in advance and will not be updated in the later iteration. 

In the process of iterative updating, the necessary conditions for the stability of sensor variable nodes have been judged first, so it is considered that the codeword sent by the sensor node has been judged accurately in the process of message iteration. Decoding the information of the sensor node will not bring a big error to the SCMA system, and in this process, the multi-node detection algorithm of the system can effectively reduce complexity.

Since the stability of sensor variable nodes in the iterative updating process is judged first in the S-T-Max-log-MPA algorithm, Equation (9) of the resource node updating process of the algorithm is modified as Equation (12):

(12) $$I_{c_{k}\to u_{j}}^{t}(x_{j})=2\times \frac{1}{\sqrt{2\pi }\delta }\times \underset{∼x_{j}}{max}\left\{-\frac{1}{2\delta^{2}}{\left\|y_{k}-\sum_{v\in \xi_{k}}h_{k,v}x_{k,v}\right\|}^{2}+\prod_{m\in \xi_{k}/j}I_{c_{m}\to u_{k}}^{t}(x_{j})+\prod_{m\in \xi_{k}/j}I_{c_{m}\to u_{k}}^{t-1}(x_{j})\right\}.$$

Then, the sensor nodes are judged by the LLR of each sensor node’s coding bit, as shown in Equations (13) and (14):

(13) $$Q(x_{j})=ap_{v}(x_{j})\times \prod_{m\in \xi_{k}/j}I_{c_{m}\to u_{k}}(x_{j}),$$

(14) $$LLR_{j,x}=log(\frac{\sum_{m:b_{m,i}=0}Q(x_{j})}{\sum_{m:b_{m,i}=1}Q(x_{j})}),$$

where, $ap_{v}(x_{j})$ represents the prior probability of user j codeword, $LLR_{j,k}$ represents the log likelihood ratio, $\sum_{m:b_{m,i}=0}Q(x_{j})$ represents the output probability of the decoded variable node, and $\sum_{m:b_{m,i}=1}Q(x_{j})$ represents the output probability of the variable node to be decoded.

## 6. Complexity Analysis

The complexity of MPA and its improved algorithms are mainly determined by the number of multipliers used in the algorithm. In the original MPA, the high complexity is mainly caused by the large amount of operations of the EXP algorithm in the iterative process, the large space occupied in the iterative updating process of the message, and the storage space occupied by the updating between the variable node and the function node. The number Q_m_ of multipliers used in the original MPA calculation is as Equation (15):

(15) $$Q_{m}=t_{max}\times K\times d_{f}\times M^{d_{f}}\times (2\times d_{f}+1)+t_{max}\times J\times d_{v}\times (d_{v}-2),$$

where d_f_ represents the number of sensor nodes per resource block and d_v_ represents the number of resource blocks per sensor node.

In the proposed algorithm, the user node stability and threshold are first used for decision, so the accuracy of user information decoding is increased, and the maximum number t′_max_ of user iterations is reduced, which is less than t_max_. Then, the user node information updating is integrated into the resource node information updating, which reduces the storage space of the intermediate information variables. Finally, in the whole operation process, the operation of logarithmic field is used to change EXP operation into addition operation. The number Q′_m_ of multipliers used in the S-T-Max-log-MPA algorithm can be calculated as Equation (16):

(16) $${Q^{\prime }}_{m}={t^{\prime }}_{max}\times K\times d_{f}^{2}\times M^{d_{f}}.$$

Therefore, the number Q′_m_ of multipliers of the proposed algorithm in this paper is greatly reduced, which is less than Q_m_. So the complexity of the detection algorithm will be greatly reduced.

## 7. BER Analysis

In the communication system, the accuracy of user information transmission is mainly measured by BER performance. In the T-MPA algorithm, because the hard decision mechanism is used to make a decision and decode the user information in advance, the correctness of information transmission is greatly reduced, the soft information is lost, and the decision of other user nodes will also be affected. This method will result in worse BER performance, especially at low threshold. In the S-MPA algorithm, because the user node information updating is integrated into the resource node information updating, the decoding and update of user information is stopped before the stability of user information is judged, which leads to incomplete transmission of user information and the loss of certain information [46]. 

In this proposed algorithm, the necessary condition of the stability of sensor nodes information is judged first, and then the sensor node information updating is integrated into the resource node information updating, which improves the stability of sensor information and reduces the BER of sensor information transmission. Especially when the threshold setting is low, the decision of the necessary condition of sensor node stability is more significant to reduce BER performance of T-MPA. Finally, the iterative update of messages is carried out with the threshold decision, which reduces the loss of information during the updating process and further improves the accuracy of sensor node information transmission.

## 8. Analysis of Simulation Results

In order to test and compare the BER performance and complexity between the S-T-Max-log-MPA algorithm and the original MPA algorithm in IWSN, simulation experiments are carried out. In the experiment, the selected parameters are J = 6, K = 4, M = 4, N = 1000. The overload factor is 150%, and the channel is a Gaussian white noise (AWGN) channel. The codebook used is a 4-dimensional codebook published by Huawei in reference [47].

### 8.1. BER Analysis

**Figure 3:**
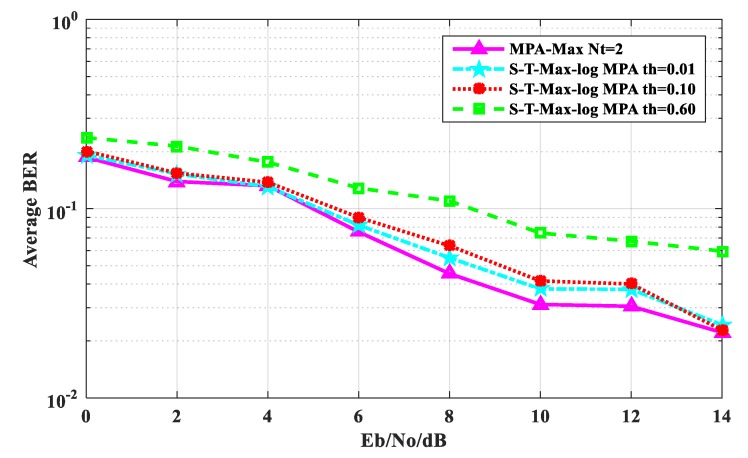
Comparison of average bit error ratio (BER) performance when t_max_ = 2.

Figure 3 shows the average BER performance comparison between the original MPA algorithm and the S-T-Max-log-MPA algorithm when the maximum number of iterations is t_max_ = 2. It can be seen from Figure 3 that the threshold value of the S-T-Max-log-MPA algorithm is smaller, and the BER of the S-T-Max-log-MPA algorithm is closer to that of the original MPA algorithm. The BER performance of thresholds th = 0.01 and th = 0.10 is similar to that of the original MPA algorithm when E_b_/N_o_ ≤ 4 dB is used. When 4 dB < E_b_/N_o_ < 14 dB, the BER performance of threshold th = 0.01 is slightly higher than that of the original MPa algorithm, by 1.834%, and that of threshold th= 0.10 is slightly higher than that of the original MPa algorithm, by 0.967%. For threshold th = 0.60, the BER performance of S-T-Max-log-MPA is 5.05% higher than that of the original MPA at E_b_/N_o_ = 0 dB and 3.864% higher than that of the original MPA at E_b_/N_o_ = 14 dB. But on the whole, the BER performance of the S-T-Max-log-MPA algorithm is good. So when t_max_ = 2, the threshold is smaller and the BER performance is better.

**Figure 4:**
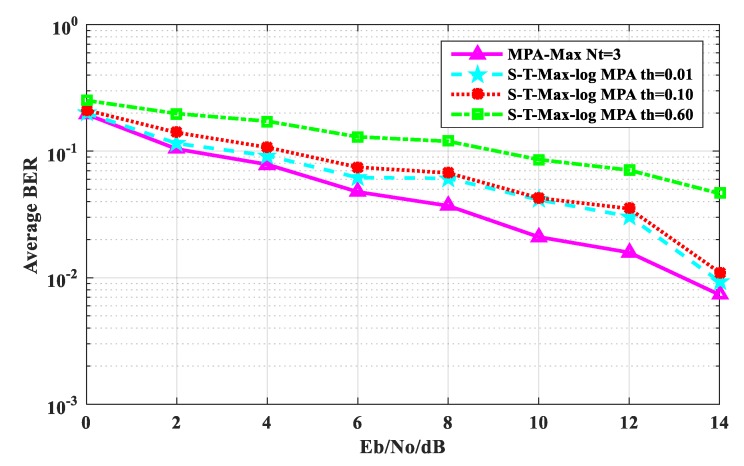
Comparison of average BER performance when t_max_ = 3.

Figure 4 shows the average BER performance comparison between the S-T-Max-log-MPA algorithm and the original MPA algorithm when the maximum number of iterations is t_max_=3. As can be seen from Figure 4, when the S-T-Max-log-MPA algorithm is E_b_/N_o_ ≤ 6 dB, the BER algorithm with threshold th = 0.01 is closest to the BER of the original MPA algorithm. When E_b_/N_o_ = 0 dB, the threshold of the algorithm is th = 0.01, the BER performance is 0.47% higher than the original MPA algorithm, and when E_b_/N_o_ = 14 dB, it is 0.20% higher than the original MPA algorithm. When E_b_/N_o_ = 0 dB, the threshold of the algorithm is th = 0. 10, and the BER performance is 1.52% higher than that of the original algorithm. At E_b_/N_o_ = 14 dB, it is 0.3667% higher than the original MPA algorithm. For threshold th = 0.60, the BER performance of the S-T-Max-log-MPA algorithm is 6.13% higher than that of the original MPA algorithm at E_b_/N_o_ = 0 dB. At E_b_/N_o_ = 14 dB, it is 3.8997% higher than the original MPA algorithm. But on the whole, the BER performance of the S-T-Max-log-MPA algorithm is close to that of the original MPA algorithm. So when t_max_ = 3 the threshold is small and the BER performance is better.

**Figure 5:**
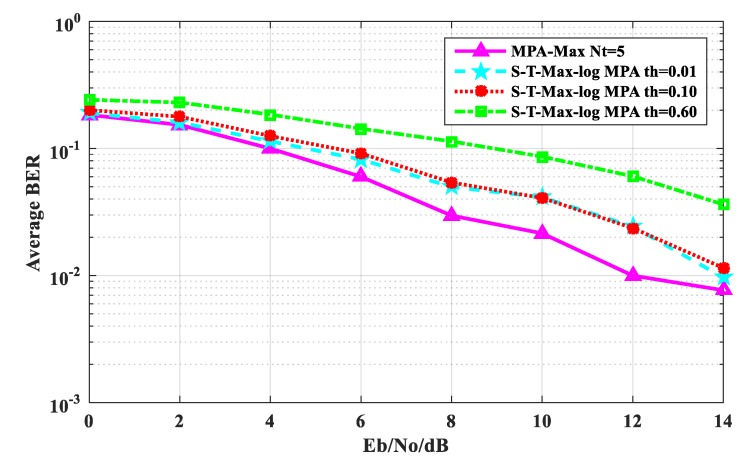
Comparison of the average BER performance when t_max_ = 5.

Figure 5 shows the comparison of the average BER performance between the S-T-Max-log-MPA algorithm and the original MPA algorithm when the maximum number of iterations is t_max_ = 5. As can be seen from Figure 5, the BER performance of the S-T-Max-log-MPA algorithm with thresholds of th = 0.01 and th = 0.10 is similar to that of the original MPA algorithm. When E_b_/N_o_ = 0 dB, the BER performance with threshold th = 0.01 is 0.65% higher than the original MPA algorithm, and the BER performance with threshold th = 0.10 is 0.3833% higher than the original MPA algorithm. At E_b_ / N_o_ = 14 dB, the BER performance of the S-T-Max-log-MPA algorithm at threshold th = 0. 01 is 0.20% higher than that of original MPA algorithm, and the BER performance at threshold th = 0.10 is 1.69% higher than that of original MPA algorithm. When the threshold is th = 0.60, the BER performance of the S-T-Max-log-MPA algorithm is 5.78% higher than that of the original MPA algorithm at E_b_/N_o_ = 0 dB. At E_b_/N_o_ = 14 dB, it is 2.8663% higher than the original MPA algorithm. On the whole, the BER performance of the S-T-Max-log-MPA algorithm is good. So when t_max_ = 5, the threshold is smaller, the BER performance is better.

**Figure 6:**
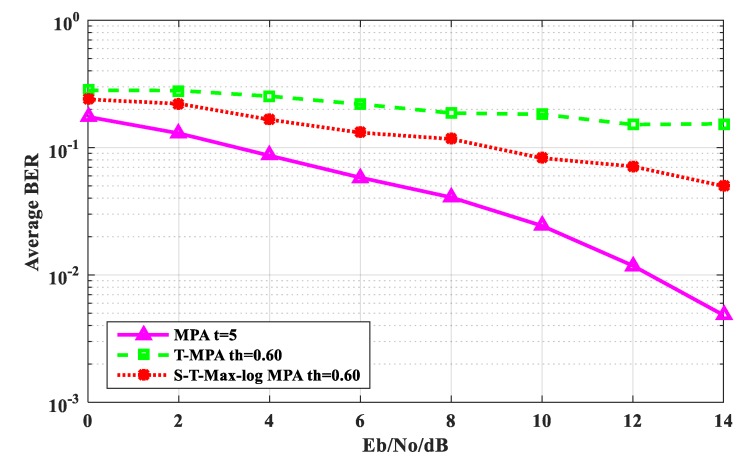
Comparison of average BER performance when t_max_ = 5 and th = 0.60.

Figure 6 shows the comparison of the average BER performance among the S-T-Max-log-MPA algorithm experiments: the original MPA algorithm and the T-MPA algorithm, in which the maximum number of iterations t_max_ = 5 and the threshold is th = 0.60. It can be seen from Figure 6 that the BER performance of the S-T-Max-log-MPA algorithm is lower than that of the T-MPA algorithm, which is 4.26% lower than that of the T-MPA algorithm when E_b_/N_o_ = 0 dB. When E_b_/N_o_ = 14 dB, it is 10.397% lower than the T-MPA algorithm. The BER performance of the S-T-Max-log-MPA algorithm is higher than that of the original MPA algorithm, which is 6.47% higher than that of the original MPA algorithm at E_b_/N_o_ = 0 dB. At E_b_/N_o_ = 14 dB, it is 4.4997% lower than the original MPA algorithm. According to the comparison results, we can see that although the BER performance of the S-T-Max-log-MPA algorithm is higher than that of the original MPA algorithm, it is lower than that of T-MPA algorithm.

### 8.2. Complexity Analysis

**Figure 7:**
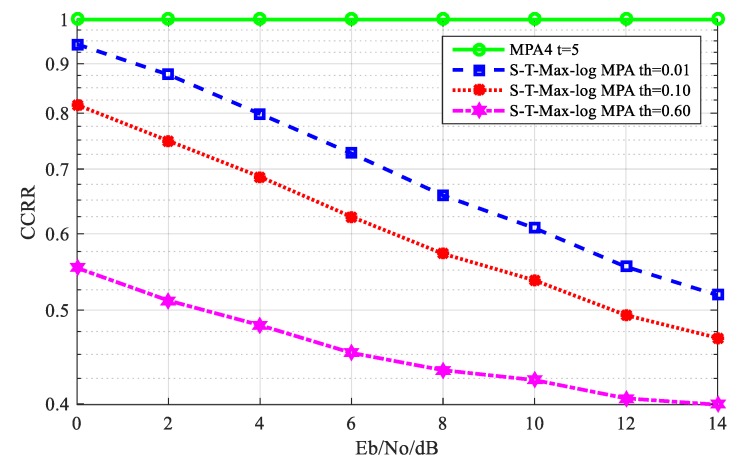
Comparison of the Computational Complexity Reduction Ratio (CCRR) between original Message Passing Algorithm (MPA) algorithm and S-T-Max-log-MPA algorithm, where S-T-Max-log-MPA is Maximum logarithm Message Passing Algorithm based on Serial and Threshold.

Figure 7 shows the comparison of the computational complexity reduction ratio (CCRR) between the S-T-Max-log-MPA algorithm and the original MPA algorithm when the maximum number of iterations is 5; the complexity reduction ratio of the algorithm is defined as Equation (17). As can be seen from Figure 7, at the threshold of th = 0.01, when E_b_/N_o_ = 0 dB, the CCRR of the S-T-Max-log-MPA algorithm is 5.74%, which is lower than that of the original MPA algorithm. When E_b_/N_o_ = 14 dB, the CCRR is 48.18% lower than the original MPA algorithm. At the threshold of th = 0.10, when E_b_/N_o_ = 0 dB, the CCRR of the S-T-Max-log-MPA algorithm is 18.42% lower than that of the original MPA algorithm. When E_b_/N_o_ = 14 dB, CCRR is 53.20% lower than the original MPA algorithm. When the threshold is th = 0.60, CCRR is more effective than the threshold values of th = 0.01 and th = 0.10. At E_b_/N_o_ = 0 dB, CCRR is 44.71% lower than the original MPA algorithm. At E_b_/N_o_ = 14 dB, CCRR is 60.10% lower than the original MPA algorithm. It can be seen from the figure that the CCRR of the S-T-Max-log-MPA algorithm under different thresholds is lower than the CCRR of the original MPA algorithm, so the S-T-Max-log-MPA algorithm can effectively reduce the CCRR of the SCMA system detection algorithm, and the effect is more obvious with the increase of E_b_/N_o_ and the threshold.

(17) $$CCRR=\frac{\mathrm{Complexity}\;\mathrm{of}\;A\;\mathrm{algorithm}}{\mathrm{Complexity}\;\mathrm{of}\;B\;\mathrm{algorithm}}$$

## 9. Conclusions

The application of 5G brings new opportunities for the development of IWSN. The IWSN composed of a large number of intelligent wireless sensor nodes has similar characteristics with the mobile communication network, and also has the problem of multi-node access and detection. Therefore, based on the research of the original MPA and its improved algorithms, the related 5G SCMA technologies are combined to carry out the research of multi-node detection in IWSN, and a novel S-T-Max-log-MPA algorithm is proposed for multi-node detection in this paper. Through the application of threshold and serial updating, the proposed algorithm reduces the iterations of sensor nodes, increases the accuracy of information transmission, and reduces the computational complexity. The simulation results show that the accuracy of information transmission of the S-T-Max-log-MPA algorithm is effectively changed, with accuracy superior to the T-MPA algorithm (up to 10.397%). The complexity of multi-node passing algorithm is effectively reduced, which is superior to the original MPA algorithm (up to 60.1%). Therefore, the S-T-Max-log-MPA algorithm can not only ensure the system bit error rate, but also effectively reduce the complexity of the multi-node detection algorithm. At the same time, it can effectively solve the serious problem that the accuracy of T-MPA algorithm decreases when the threshold value is low.
